# The interplay between physical exercise and autophagy signaling in brain health, neurodegenerative diseases and aging

**DOI:** 10.3389/fnagi.2025.1579208

**Published:** 2025-07-29

**Authors:** Bo Gao, Li Wang, Jian Gong, Zehua Zhu, Qi Liu, Han Yuan, Haitao Wang

**Affiliations:** 1 Department of Physical Education, Liaocheng University, Liaocheng, China; 2 Department of Physical Education and Sport, Shanghai Ocean University, Shanghai, China; 3 Department of Physical Education, Graduate School, Pukyong National University, Busan, Republic of Korea; 4 Major in Sport Science, Hanyang University, Seoul, Republic of Korea; 5 Department of Physical Education, Shanghai Jiaotong University, Shanghai, China; 6 Department of Physical Education, Kyungpook National University, Daegu, Republic of Korea

**Keywords:** exercise, autophagy, brain, neurodegenerative diseases, health

## Abstract

Brain health is increasingly recognized as a critical component of overall wellbeing, particularly concerning neurodegenerative diseases, which are characterized by the progressive degeneration of the nervous system. Conditions such as Alzheimer’s disease (AD) and Parkinson’s disease, together with less common disorders, resembling Amyotrophic lateral sclerosis (ALS), and Huntington’s disease (HD), significantly impact cognitive and physical health, affecting over 50 million individuals worldwide. This review explores the multifaceted relationship between brain health and neurodegeneration, emphasizing the roles of biological, environmental, and lifestyle factors. Notably, physical activity has been identified as a potent intervention that enhances neuroplasticity and metabolic resilience while mitigating the effects of neurodegeneration. Research indicates that exercise activates autophagy, which is crucial for clearing neurotoxic aggregates like amyloid-beta and α-synuclein, thereby promoting neuronal health. Additionally, exercise stimulates the production of neurotrophic factors such as BDNF and GDNF, which are essential for neuronal survival and function. Despite the promising findings regarding exercise as a preventive and therapeutic strategy for neurodegenerative diseases, further investigation into the underlying mechanisms is necessary to optimize these interventions. This review aims to elucidate the complex interactions between exercise, autophagy, and brain health to provide insights into effective strategies for combating neurodegeneration.

## 1 Introduction

The importance of maintaining brain health has become increasingly evident, especially when considering neurodegenerative disorders. These conditions, which cause progressive nervous system deterioration, create substantial obstacles for both mental and physical functioning. Multiple components, including biological mechanisms, environmental conditions, and daily habits, influence the relationship between brain wellness and neurodegeneration (Ayeni et al., 2022; Bongioanni et al., 2021). Neurodegenerative conditions represent various long-term illnesses that gradually impair nervous system function, with the brain being particularly vulnerable. Major examples include Alzheimer’s, Parkinson’s, ALS, and Huntington’s disease. These permanent and currently untreatable conditions typically emerge in later years, affecting more than 50 million individuals worldwide and severely impacting both longevity and life quality (Jiang et al., 2025; Soo et al., 2020).

Brain wellness encompasses both cognitive capabilities and overall health, shaped by various external influences. Taking a comprehensive view of brain health requires considering multiple approaches that incorporate biological processes, personal habits, and social-psychological elements (Lamptey et al., 2022; Mintzer et al., 2019). Studies indicate that specific lifestyle modifications can support brain function and potentially reduce neurodegeneration’s impact. Physical exercise, in particular, has been shown to enhance brain plasticity and metabolic strength. Recent studies point to disrupted neuronal autophagy as a potential trigger for neurodegeneration in Alzheimer’s disease. This disruption leads to amyloid-beta accumulation, plaque development, immune cell activation, inflammation, and eventual nerve cell death (Halma et al., 2024; Popescu et al., 2025). The process is further complicated by tau protein aggregation, forming tangles that impair cellular function. Genetic research has identified connections between autophagy-related genes and increased amyloid presence. Similarly, in Parkinson’s disease, autophagy plays a vital role in managing α-synuclein buildup within Lewy bodies, demonstrating its significance across various neurodegenerative conditions (Lu et al., 2023; Santiago and Potashkin, 2023).

While aging and neurodegeneration can impair autophagy function, leading to cellular imbalance, research suggests that exercise can activate autophagy mechanisms. This activation helps prevent age-related decline and slow neurodegenerative processes, particularly benefiting older adults. The complex interplay between brain wellness and neurodegeneration highlights the need for diverse approaches to understanding and enhancing cognitive health (Andreotti et al., 2020; Guo et al., 2018). With ongoing discoveries about underlying biological processes and the recognized benefits of lifestyle factors, particularly exercise, taking proactive steps toward brain health becomes crucial. This approach could enhance individual wellbeing while reducing the societal impact of neurodegenerative conditions (Pang et al., 2019; Wu et al., 2024). Current review aims to explore how exercise, autophagy, and brain health interconnect at the molecular level, providing deeper insights into exercise’s effects on brain function.

## 2 Neuronal autophagy: a key cellular mechanism in brain health

**FIGURE 1:**
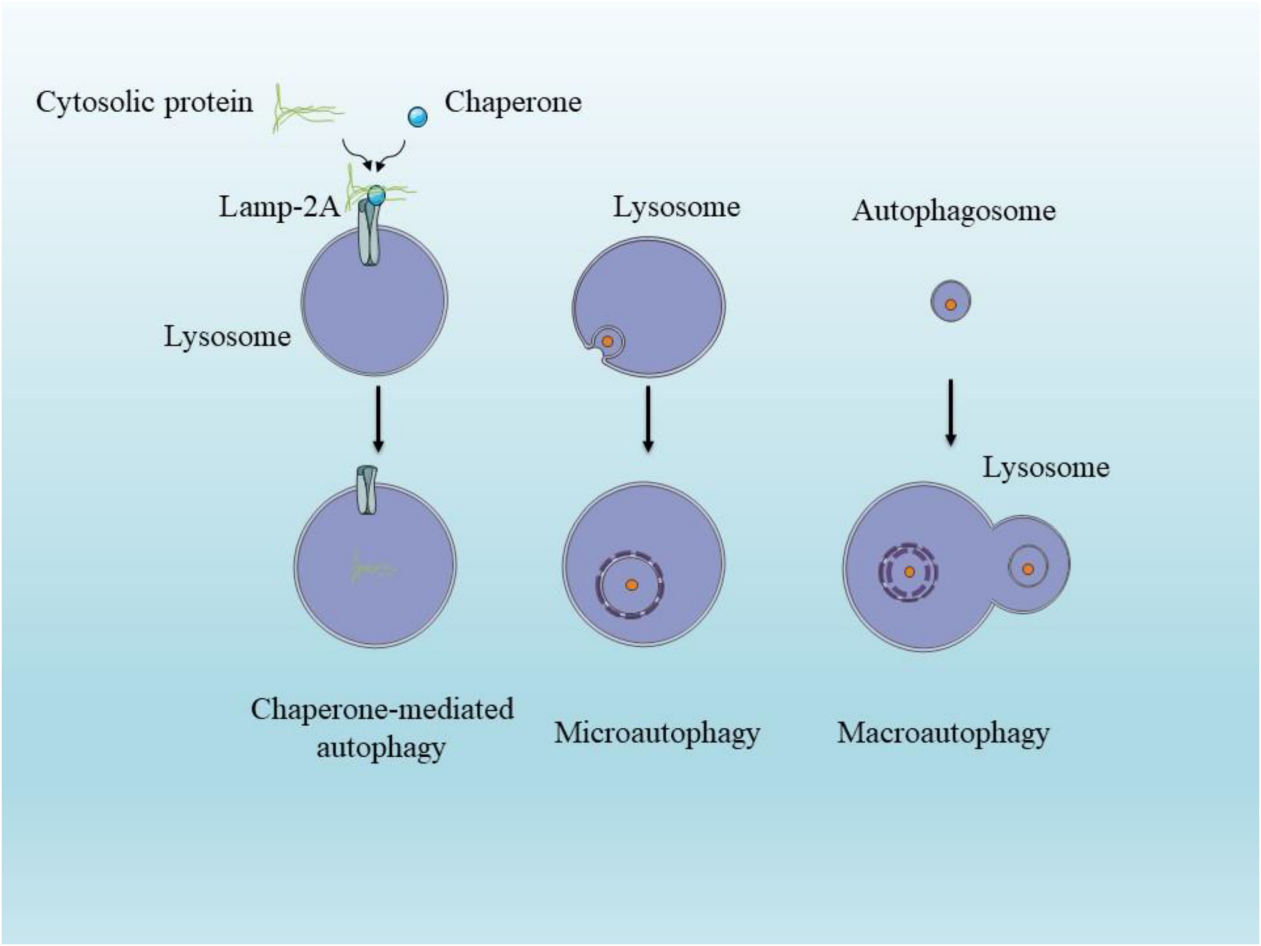
Illustration of the three major autophagy pathways in neurons. Macroautophagy involves sequestration of cytosolic cargo into autophagosomes that fuse with lysosomes. Microautophagy occurs via direct lysosomal membrane invagination. Chaperone-mediated autophagy selectively translocates soluble proteins through LAMP2A channels with the help of HSC70 chaperones.

Cells maintain their health through a cleanup and reuse mechanism called neuronal autophagy. This process involves capturing unnecessary cellular components and directing them to specialized structures like lysosomes and vacuoles for breakdown and disposal. In mammals, this vital process takes three distinct forms: macroautophagy, microautophagy, and chaperone-mediated autophagy, with macroautophagy being the most extensively studied and commonly referenced when discussing autophagy (Dashti et al., 2024; Figure 1). Macroautophagy serves two key functions: it helps maintain normal cell balance during growth and development, and it activates during challenging conditions like nutrient scarcity or energy shortages (Kiani et al., 2025; Son et al., 2012; Wang L. et al., 2019). The process unfolds in four distinct phases: initiation, creation of the autophagosome, combination with lysosomes to form autophagolysosomes, and finally, the breakdown and reuse of captured materials. The sequence begins when a structure called a phagophore surrounds damaged cell components or unwanted materials. This structure expands to create a double-walled compartment known as an autophagosome. This vessel then merges with a lysosome, forming an autolysosome or autophagic vacuole that breaks down the captured contents. The resulting basic components return to the cell’s interior for reuse in various metabolic processes. In contrast, microautophagy involves a more direct approach, where lysosomes directly engulf small portions of cellular material (Stavoe and Holzbaur, 2019; Wang L. et al., 2019). The third type, CMA, operates more selectively, targeting only proteins marked with a specific amino acid sequence (KFERQ). These proteins reach the lysosome through a coordinated effort between two proteins: LAMP2A and heat shock cognate 70 (HSC70) (Alfaro et al., 2019).

### 2.1 Autophagy in neuronal maintenance and brain function

**FIGURE 2:**
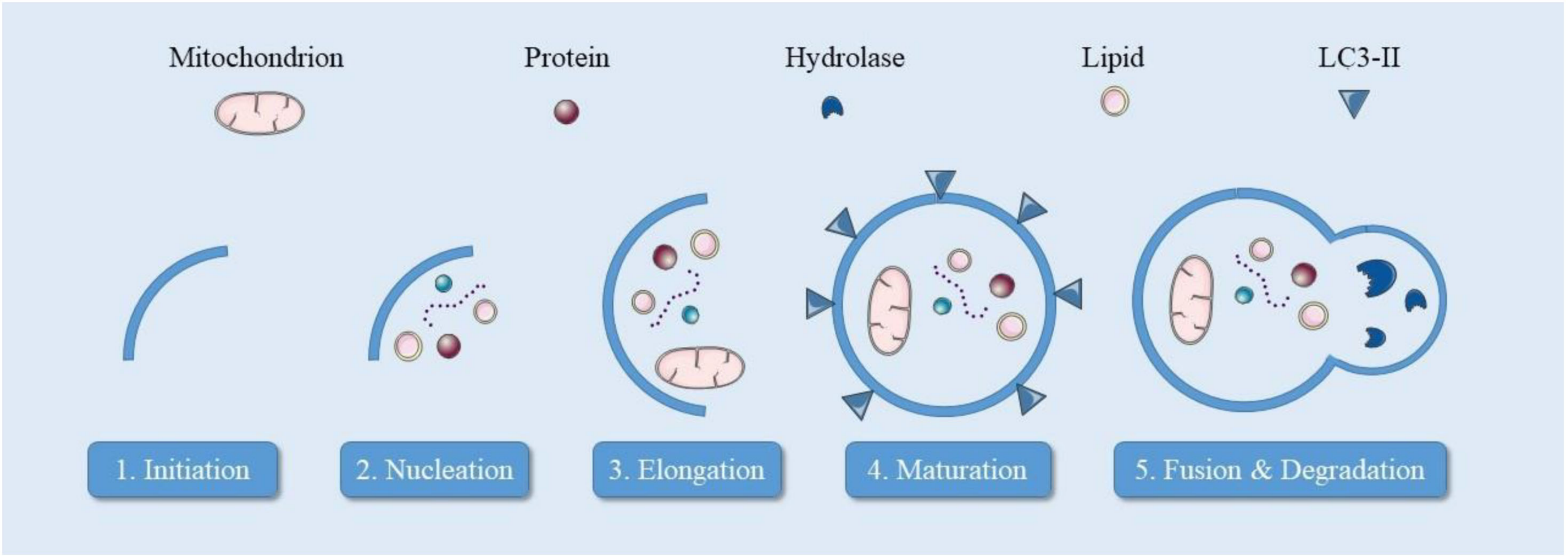
Sequential stages of macroautophagy in neurons. Initiation begins with AMPK activation and mTORC1 inhibition, leading to phagophore formation. The nucleation and elongation phases involve the Beclin1 complex and LC3-II recruitment. Mature autophagosomes are transported and fuse with lysosomes for cargo degradation and recycling. Disruptions in any of these steps are implicated in neurodegenerative diseases.

The central nervous system (CNS) is built from two main components: neurons and glial cells. Neurons serve as the operational components, designed to accept, analyze, and convey both stimulatory and inhibitory signals. Their distinctive structure includes specialized regions—dendrites and axons—reflecting their highly organized nature. Unlike most cells, neurons cannot divide after maturation, making their maintenance particularly crucial for CNS function. To maintain their complex structure and operations, neurons require active biochemical processes to recycle aging or impaired components, including mitochondria, endoplasmic reticulum, and protein clusters (Desai-Chowdhry et al., 2022; Desai-Chowdhry et al., 2023). This maintenance primarily occurs through autophagy, especially macroautophagy. This process is vital for neurons because their inability to divide means they must efficiently remove accumulated proteins to prevent toxicity. Autophagy also helps defend against pathogens and maintains proper synaptic function, as demonstrated by studies showing that disrupting autophagy through ATG5 deletion leads to endoplasmic reticulum accumulation, calcium imbalances, and altered neurotransmission. Neuronal macroautophagy proceeds through several steps (Figure 2). Initially, the ULK1/ULK2 complex triggers the process, activating Beclin1 and Ambra1 to form the Vps34 complex. This complex generates PI3P, recruiting specific proteins for phagophore formation. ATG9 protein, cycling between endosomes and Golgi, also contributes to this process. The ATG5-12-16 L1 complex and LC3II are crucial for autophagosome formation and cargo selection (Damme et al., 2015; Xi et al., 2016). In neurons, autophagic vesicles form in axons and move bidirectionally, facilitated by dynein and kinesin proteins. These vesicles eventually fuse with lysosomes, forming autolysosomes where cargo degradation occurs through acidification and enzyme activity. This process’s regulation involves mTORC1, which responds to nutrient levels. During nutrient scarcity, AMPK activates autophagy, while abundant nutrients activate mTORC1, suppressing autophagy. Disrupted mTOR signaling appears in conditions like Parkinson’s and Alzheimer’s diseases, where excessive mTOR activity reduces autophagy and promotes protein accumulation. Neurons rely exclusively on oxidative metabolism, making mitochondrial health crucial. Mitophagy, distinct from general autophagy, specifically removes damaged mitochondria. This process begins when PINK1 stabilizes on damaged mitochondria, leading to Parkin recruitment through phosphorylated Mfn2 and ubiquitin. Parkin then activates proteins like OPTN and SQSTM/p62, initiating mitophagy. This process typically occurs in the cell body and is essential for preventing oxidative stress and neurodegeneration caused by damaged mitochondria (Table 1; Kim and Guan, 2015; Valencia et al., 2021; Wang et al., 2020). Autophagy plays a crucial role in mitigating several hallmarks of aging by maintaining cellular homeostasis and promoting longevity.

**TABLE 1:** Key proteins and their roles in autophagy and mitophagy.

Protein	Role in autophagy/mitophagy	Function in neuronal health	References
ULK1/ULK2	Initiate autophagosome formation by phosphorylating downstream proteins	Regulates initiation of autophagy, essential for recycling damaged organelles	(Sun, 2016; Kim et al., 2011)
Beclin1	Part of the Vps34 complex, essential for phagophore formation	Facilitates autophagosome nucleation	(Sun, 2016)
ATG5-12-16L1 Complex	Involved in phagophore elongation and autophagosome maturation	Promotes lipidation of LC3 to LC3-II, crucial for cargo recognition	(Runwal et al., 2019)
LC3-II	Binds to autophagic substrates and mediates selectivity of cargo	Marker of autophagosomes, crucial for cargo binding and autophagosome movement	(Galluzzi et al., 2017)
mTORC1	Central regulator that inhibits autophagy under nutrient-rich conditions	Balances cellular growth and autophagy, essential for neuronal homeostasis	(Kim et al., 2011; Shang et al., 2011)
PINK1	Detects damaged mitochondria and initiates mitophagy by recruiting parkin	Ensures removal of dysfunctional mitochondria, prevents oxidative stress	(Lazarou et al., 2015)
Parkin	E3 ubiquitin ligase that tags damaged mitochondria for degradation	Facilitates mitophagy, protects neurons from ROS-induced damage	(Lazarou et al., 2015)
OPTN & SQSTM/p62	Receptor proteins that recognize ubiquitinated mitochondria and facilitate their engulfment by autophagosomes	Essential for selective autophagy and mitophagy	(Lazarou et al., 2015)

Autophagy aids in DNA repair mechanisms by degrading damaged nuclear components and reducing the accumulation of genotoxic stressors. It also helps maintain chromatin integrity, potentially minimizing mutations that accelerate aging. Autophagy supports telomere maintenance by reducing oxidative damage, which is a major contributor to telomere shortening. It indirectly influences telomerase activity, helping to stabilize telomere length. Autophagy contributes to chromatin remodeling and the clearance of dysfunctional histones, which may help regulate gene expression patterns associated with aging. By degrading misfolded proteins and damaged organelles, autophagy plays a vital role in preventing protein aggregation, a hallmark of aging-related neurodegenerative diseases (Esclatine et al., 2009). Autophagy is intricately linked to nutrient-sensing pathways, including AMPK and mTOR. Activation of autophagy enhances metabolic adaptation, mimicking effects of caloric restriction a well-known longevity-promoting intervention. Mitophagy, a specialized form of autophagy, selectively removes damaged mitochondria, preserving energy efficiency and reducing oxidative stress, which contributes to aging. Autophagy helps eliminate senescent cells, preventing their accumulation, which otherwise promotes inflammation and tissue dysfunction. Autophagy supports stem cell renewal and function by maintaining a healthy intracellular environment, reducing damage accumulation, and ensuring proper differentiation capacity (Kiani et al., 2025). By degrading secreted inflammatory mediators, autophagy may regulate extracellular vesicle release and mitigate age-associated changes in cellular signaling. Autophagy plays a key role in limiting excessive inflammatory responses by degrading damaged organelles and pro-inflammatory mediators, contributing to systemic anti-aging effects. Autophagy influences gut microbiome balance by regulating immune responses to bacterial pathogens, ensuring intestinal barrier integrity, and promoting a healthy microbiome composition (Randall-Demllo et al., 2013; Singh, 2010).

### 2.2 Dysregulation of autophagy in neurodegenerative diseases

**FIGURE 3:**
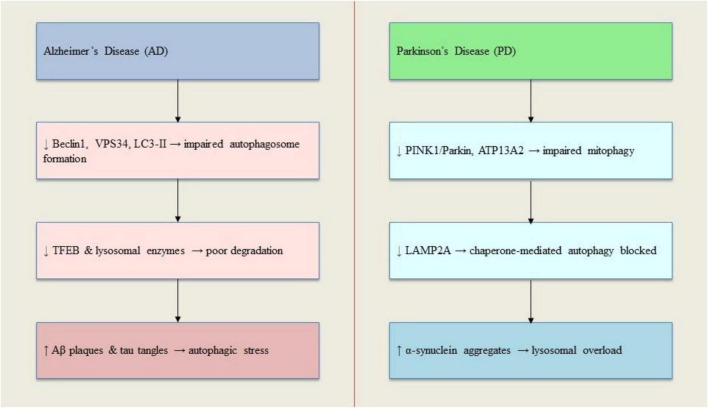
Disruption of autophagy in Alzheimer’s and Parkinson’s diseases. In AD, autophagosome formation and lysosomal degradation are impaired, leading to Aβ and tau accumulation. In PD, defects in mitophagy-related proteins (PINK1, Parkin) and CMA components (LAMP2A) contribute to α-synuclein buildup and mitochondrial dysfunction. Both conditions involve defective autophagosome–lysosome fusion and lysosomal clearance failure.

Neurodegenerative conditions like Alzheimer’s and Parkinson’s diseases are characterized by irregular protein aggregation and impaired protein degradation mechanisms. When autophagy becomes compromised, it fails to clear proteins like α-synuclein in Lewy bodies, and beta-amyloid and tau in amyloid plaques and neurofibrillary tangles (Figure 3). Research shows α-synuclein accumulation in dopamine-producing neurons of Parkinson’s patients and experimental models. Studies using α-synuclein lentivirus revealed increased autophagy markers and Beclin1, suggesting disrupted protein clearance, while mTOR inhibition through rapamycin showed therapeutic potential (Fields et al., 2019; Tsunemi et al., 2020). Various genetic factors influence the interplay between α-synuclein buildup, autophagy-lysosome dysfunction, and mitophagy disruption. These include rare familial mutations (A30P, A53T, PINK1/Parkin, LRRK2) and risk genes like cathepsin B and D. The A30P and A53T mutations enhance protofibril formation, compromising lysosomal function. Studies in neuroblastoma cells showed α-synuclein overexpression affects calcium signaling and lysosomal properties. The LRRK2 G20195 mutation impairs autophagosome movement in neurons, while PINK1 and Parkin mutations disrupt mitochondrial maintenance and calcium balance. In Alzheimer’s disease, evidence from patient samples, stem cell-derived neurons, and animal models points to widespread disruption of cellular cleanup mechanisms, affecting amyloid precursor protein processing and tau regulation (Boecker et al., 2021; Calabresi et al., 2023). The SORL1 gene, crucial for protein trafficking, influences late-onset Alzheimer’s risk. Its absence leads to enlarged endosomes, increased amyloid production, and disrupted autophagosome formation. Reduced Beclin levels in Alzheimer’s patients further highlight autophagy’s importance. Beta-amyloid oligomers create dystrophic neurites early in Alzheimer’s progression. ATG9A, an autophagy initiator protein, accumulates in these structures near amyloid plaques. The endosomal system, regulated by the retromer complex (including VPS35, VPS29, VPS26), significantly influences neuronal autophagy. Retromer dysfunction contributes to both Alzheimer’s and Parkinson’s through protein aggregate accumulation. Research shows VPS35 deficiency impairs cellular cleanup mechanisms and promotes protein aggregation, including tau buildup in brain endothelial cells (Ayeni et al., 2022; Polemiti et al., 2024).

## 3 Physical exercise and brain health

Recent years have seen growing evidence linking regular exercise to enhanced cognitive function in older adults, complementing its known advantages for reducing death rates, illness, and improving physical capabilities. This connection is particularly significant as people aged 65 and above constitute the most rapidly expanding age group globally. Projections indicate that within a decade, the United States will witness an unprecedented demographic shift, with seniors making up 20% of citizens and exceeding the child population for the first time. Research shows that aging adults consistently rank cognitive health as their primary health concern, viewing it as essential for maintaining self-sufficiency (Quigley et al., 2020; Xu et al., 2023). While it is promising that exercise habits can promote healthy brain aging, precise prescriptions are needed to effectively use exercise as medicine. The revised Physical Activity Guidelines for Americans acknowledges exercise’s role in brain health and advocates for increased movement and reduced sedentary behavior. However, research methods and findings vary considerably regarding exercise specifications. There’s currently no agreement on the ideal exercise type or amount for maximizing cognitive benefits, though a comprehensive analysis revealed that total exercise duration (approximately 52 h) was the key factor in cognitive enhancement. This review also noted that exercise primarily improved executive functioning and information processing speed—cognitive areas typically showing early age-related decline (Erickson et al., 2019; Zhang et al., 2020). Although this initial dosage research represents progress, many findings about exercise’s structural and molecular effects—thought to drive cognitive improvements—come from rodent studies, raising questions about human applicability. Exercise has been shown to combat age-related brain tissue deterioration, enhance hippocampal blood supply and neural connections, boost synaptic adaptability, and stimulate important neuronal growth factors. However, uncertainty remains about which exercise types and amounts trigger these effects. Furthermore, limited research on aging animals complicates our understanding of how these findings relate to human aging processes (Chieffi et al., 2017; Gomez-Pinilla and Hillman, 2013). The study demonstrated that aerobic exercise training increases brain volume in aging humans, particularly in gray and white matter regions. The findings suggest that cardiovascular fitness is associated with preserving brain tissue, reinforcing the role of exercise in maintaining cognitive function (Colcombe et al., 2006). The research explored the impact of daily household physical activity on brain volume in older adults. Unlike recreational exercise, household activities were positively correlated with gray matter volume, particularly in the hippocampus and frontal lobe. The study suggests that even non-recreational movement contributes to brain health (Colcombe et al., 2006). This review examined the influence of physical activity on brain aging and dementia. It highlighted that exercise may reduce cognitive decline and improve neuroimaging biomarkers associated with aging. However, methodological challenges in comparing studies were noted, emphasizing the need for more standardized trials (Lautenschlager et al., 2012).

**FIGURE 4:**
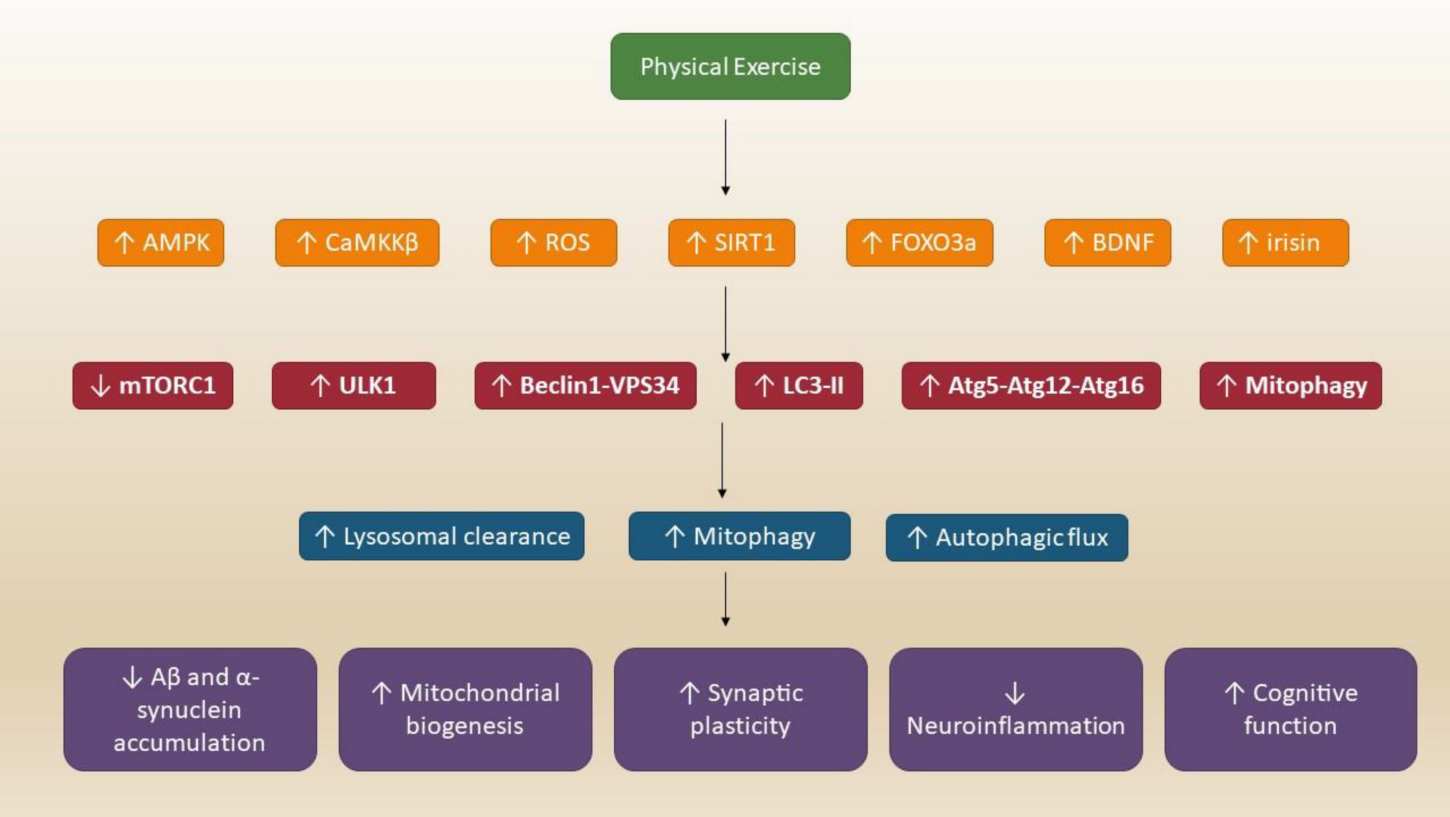
Physical exercise activates autophagy in the brain through multiple converging pathways. AMPK, CaMKKβ, and SIRT1 suppress mTORC1 and activate ULK1 and Beclin1, leading to enhanced autophagosome formation and mitophagy. This results in clearance of toxic proteins, improved mitochondrial function, and enhanced neuroplasticity-providing protection against neurodegenerative–processes.

The study investigated the effects of resistance exercise training on skeletal muscle aging. It found that exercise reduces ATF4-activated and senescence-associated mRNAs, suggesting that resistance training mitigates age-related muscle deterioration and enhances mitochondrial function (Von Ruff et al., 2025). The paper mapped the multi-omic changes in mitochondrial activity across various rat tissues following endurance training. It identified tissue-specific adaptations, including increased oxidative proteins in striated muscles and metabolic shifts in the liver and adrenal glands (Amar et al., 2024). The study analyzed transcriptomic and epigenomic signatures in response to exercise training. It found that exercise-induced molecular changes are highly tissue-specific, with distinct regulatory landscapes influencing gene expression across different organs (Nair et al., 2024). The large-scale study examined the multi-organ molecular response to endurance exercise. It provided a comprehensive atlas of how exercise modulates immune, metabolic, and stress-response pathways, offering insights into the systemic benefits of physical activity (Adkins and Bodine, 2024). These papers collectively underscore the broad physiological and molecular benefits of exercise, from brain health and cognitive preservation to muscle adaptation and systemic metabolic regulation (Figure 4).

### 3.1 Exercise-induced benefits: from brain architecture and neurogenesis to neurovascular perfusion

#### 3.1.1 Brain architecture

Brain aging is characterized by specific tissue loss, predominantly affecting the prefrontal cortex and medial temporal regions. Around age 50, healthy adults typically experience annual hippocampal shrinkage of 1–2%. These volume reductions correlate with declining memory and executive function abilities. Researchers have long suggested that exercise might help counteract these age-related changes, potentially explaining cognitive improvements in older adults who exercise regularly. Studies examining brain structural changes show varying results based on intervention duration and exercise type. Brief (12-week) moderate aerobic programs showed no significant whole-brain gray matter changes, though some participants who improved fitness and hippocampal blood flow showed localized volume increases in the hippocampal head. Longer interventions (6 months) demonstrated more substantial regional brain changes. Both high and low-intensity aerobic activities increased hippocampal density and volume (O’Shea et al., 2016; Xiao et al., 2023). Various exercise types, from Nordic walking to gymnastics, enhanced gray matter volume in prefrontal and cingulate areas. Some studies found increased volume in regions controlling attention and memory, while others showed no thickness changes despite cognitive improvements. These mixed findings suggest either longer interventions may be necessary for structural changes, or that cognitive benefits might stem from other physiological mechanisms. Different exercise types appear to affect brain structure uniquely. Dance training increased frontal and temporal cortical volume, while resistance and flexibility training enhanced occipital and cerebellar regions. The novelty and attention demand of dance may explain its broader effects compared to repetitive exercises. Extended interventions (40 weeks to 2 years) revealed interesting patterns. Tai chi and social interaction increased whole-brain volume more than light aerobic exercise, possibly due to their cognitive engagement requirements. Resistance training showed complex effects, with some studies finding decreased whole-brain volume but improved executive function (Erickson et al., 2014; Rehfeld et al., 2018). Long-term aerobic and coordination exercises differently affected hippocampal regions, with aerobic activity primarily benefiting the left hippocampus (verbal memory) and coordination training enhancing the right hippocampus (spatial memory). Regarding brain function, aging typically impairs neural network activation and communication between brain regions. Exercise interventions of various durations have shown potential to improve neural efficiency and connectivity patterns. Short-term programs (12–16 weeks) enhanced hippocampal connectivity and reduced unnecessary neural activation during memory tasks. Medium-term interventions (6 months) improved attention network efficiency, while long-term programs showed exercise-specific connectivity changes in networks crucial for executive control and memory. Different exercise modes appear to engage distinct neural pathways while achieving similar cognitive benefits. For example, aerobic exercise and coordination training both improved cognitive performance but showed different patterns of brain activation. These findings suggest that various exercise types may enhance cognitive function through different neurological mechanisms (Erickson et al., 2011; Prathap et al., 2021).

#### 3.1.2 Neurogenesis and synaptogenesis

Research with animal models helps scientists understand microscopic brain changes from exercise, particularly the development of new neurons and synaptic connections in the hippocampus. While early research strongly linked exercise to neuron formation, age significantly impacts these processes. For example, 9-month-old rodents generate hippocampal neurons at half the rate of 6-week-old ones, with further reduction to 17% by age 24 months. However, exercise can still boost neuron formation in older animals. Nineteen-month-old rodents showed 50% increased neurogenesis and 20% more glia cell formation after 6 weeks of voluntary wheel running, accompanied by better spatial learning. Remarkably, older exercising mice showed similar new cell numbers and structures as young mice, suggesting exercise enhanced their ability to convert precursor cells into neurons—a threefold increase compared to sedentary aged mice (Parnow et al., 2023; Voss et al., 2013; Wu et al., 2023). Short exercise periods (10–28 days) typically increased cell division related to neurogenesis, though one study found reduced neuron formation in very old mice after 2 weeks of exercise. Neural stem cells (NSCs), crucial for brain regeneration, decline with age—dropping by 70% at 18 months and 90% at 24 months in mice. Three weeks of exercise helped maintain NSC production in 18-month-old mice but showed limited effect in 24-month-old mice, though some studies found brief exercise periods could stimulate hippocampal NSC growth even in very old mice. Exercise appears to support neurogenesis through various mechanisms, including restoring age-depleted enzymes, counteracting harmful bacterial toxins, and reducing inflammation-related immune cells that might impair plasticity. While aging also reduces synapse formation, research on exercise’s impact on this process in older animals is limited, though young rodents show increased synaptic markers with both aerobic and non-aerobic exercise. Translating these findings to humans requires careful consideration. Recent human brain tissue studies suggest neurogenesis may continue into the eighth decade of life, maintaining similar levels from ages 14 to 80. However, older adults show decreased blood vessel formation, reduced neuroplasticity markers, and diminished capacity for generating new neurons, which might explain some age-related cognitive changes. These findings suggest that while the aging brain maintains some capacity for generating new neurons, other factors may contribute to cognitive decline (Daynac et al., 2016; Klein et al., 2017; Liu et al., 2020; Yang et al., 2015).

#### 3.1.3 Synaptic adaptability

Synaptic neuroplasticity refers to enduring modifications in how effectively synapses communicate, specifically through processes called long-term potentiation (LTP) and long-term depression (LTD). Scientists first discovered these mechanisms by repeatedly stimulating hippocampal neurons electrically and measuring their responses with precise electrode placement, revealing how these processes contribute to memory formation. Currently, LTP stands as the primary explanation for brain-wide synaptic activity involved in both cognitive and motor learning. Animal studies have shown that aging diminishes LTP capacity (Abraham et al., 2019; Dringenberg, 2020). In young rodents, regular physical activity enhances visuospatial abilities, which correlates with stronger LTP in hippocampal neurons. Exercise has also been shown to restore LTP function and improve cognition in various young rodent models where LTP was initially impaired. Research specifically examining exercise’s effects on LTP in aged rodents is limited but promising. Two key studies demonstrated enhanced synaptic plasticity following exercise programs. A 12-week program improved LTP and enhanced performance in water maze navigation and object recognition tasks. Another study, starting in middle age and continuing for 8 months, found that sustained aerobic exercise prevented age-related LTP decline and maintained spatial learning abilities. These findings suggest that both shorter and longer exercise interventions can positively impact synaptic plasticity in aging brains (Baek, 2016; Patten et al., 2015).

#### 3.1.4 Neurovascular perfusion

Research indicates that regular physical activity helps maintain and enhance blood flow to the brain, supporting cognitive function. Studies show that from middle age to later years, overall cerebral blood flow typically diminishes by about 30%, correlating with brain tissue loss and reduced metabolic activity. Exercise leads to both enhanced cardiac output and blood redistribution, responding to increased PaCO2 and greater blood requirements in active muscles. Brain blood vessels also adapt, though less markedly. Blood flow to the brain increases gradually with exercise intensity until reaching 60% VO2max, after which it stabilizes and eventually returns to baseline at higher intensities (Alfini et al., 2019; Liu et al., 2023). Researchers use measurements of middle cerebral artery velocity (MCAv) to understand brain blood flow regulation. While older adults show reduced MCAv both at rest and during exercise, the proportional increase during physical activity remains comparable between younger and older individuals. The lower MCAv observed with aging might serve as a natural adjustment to higher blood pressure and reduced vessel elasticity. Furthermore, both age groups show similar improvements in blood vessel responsiveness to carbon dioxide after 3 months of moderate to vigorous aerobic training. Research examining the effects of 12–16-week moderate aerobic exercise programs on brain blood flow has produced varying results. One study found increased blood flow in the anterior cingulate cortex compared to non-exercising controls, while another showed no significant changes when measured against cognitive training participants. Similarly mixed findings emerged regarding hippocampal blood flow: a 12-week program showed no improvement versus stretching exercises, but a 16-week study involving older participants with memory concerns demonstrated enhanced hippocampal blood flow compared to those doing light stretching and receiving education (Fisher et al., 2008; Kleinloog et al., 2022; Tomoto et al., 2023).

## 4 Interplay between physical exercise and autophagy

**FIGURE 5:**
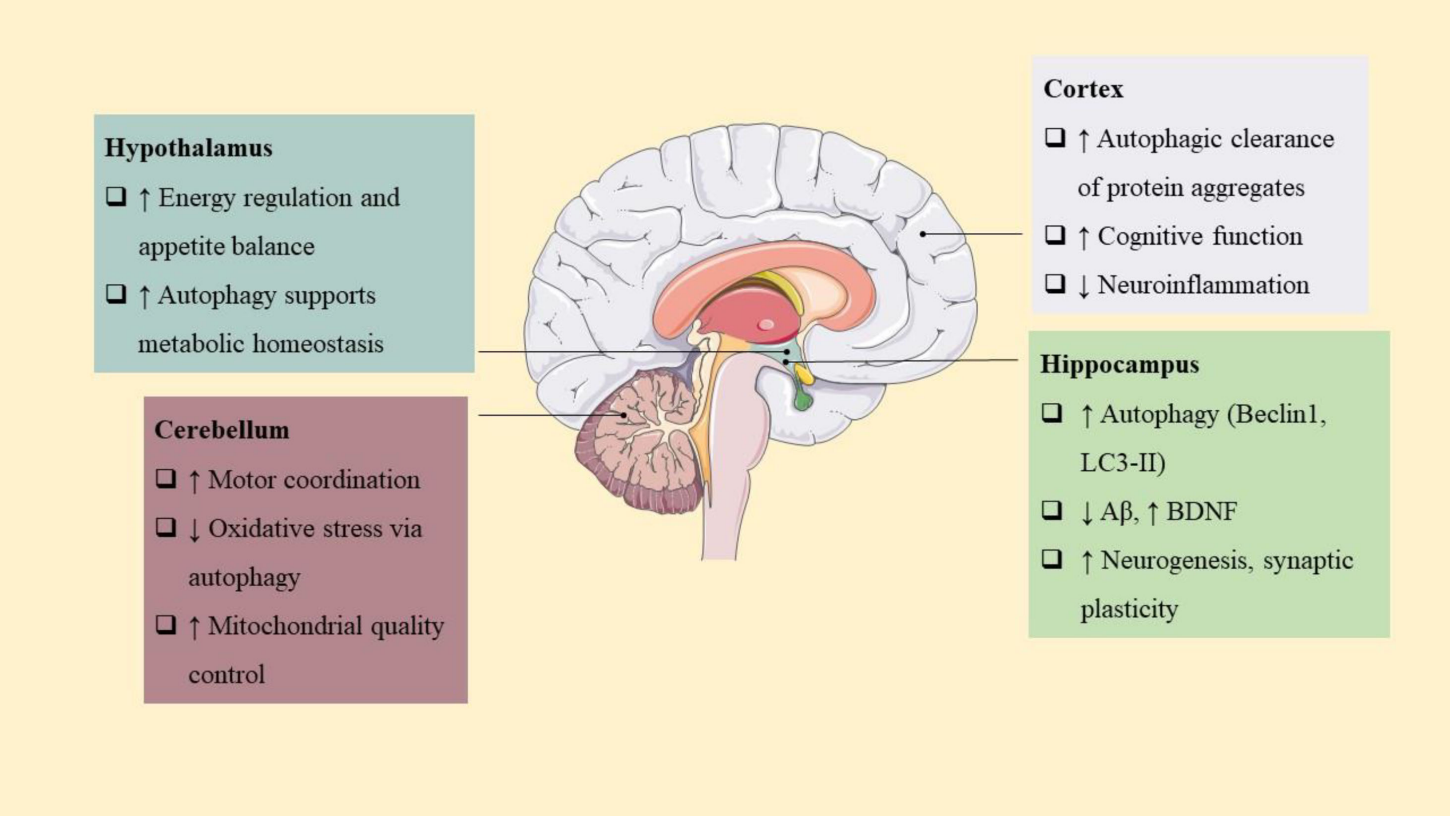
Exercise-induced autophagy enhances brain health through region-specific mechanisms. In the hippocampus and cortex, autophagy promotes neurogenesis, protein clearance, and cognitive function. In the substantia nigra, it supports mitophagy and dopaminergic neuron survival. Other regions like the cerebellum and hypothalamus benefit via improved motor control and energy regulation. These effects underlie the neuroprotective potential of physical activity in aging and neurodegeneration.

The scientific understanding of exercise-triggered autophagy, though first noted in 1984, has gained significant research momentum only recently (Figure 5). A breakthrough study in 2011 by Grumati et al. revealed that physical activity triggers autophagy in muscle tissue, evidenced by LC3-I to LC3-II conversion and autophagosome formation. Their research showed that mice lacking collagen VI, which impairs autophagy function, experienced increased muscle deterioration and cell death during exercise. Exercise creates various cellular stresses, including disrupted calcium balance, increased oxidative stress from heightened mitochondrial activity, and altered electrolyte concentrations. These changes can compromise cell function and survival. Autophagy helps maintain cellular health post-exercise by removing damaged cellular components and potentially supporting energy balance by providing amino acids as alternative fuel sources (Grumati et al., 2011; Rocchi and He, 2017). Research using BCL2AAA transgenic mice, which lack exercise-induced autophagy despite normal baseline levels, demonstrated autophagy’s role in exercise metabolism. These mice showed altered glucose regulation and reduced exercise capacity compared to normal mice. Studies suggest autophagy mediates exercise-related improvements in insulin sensitivity, though some research presents conflicting findings regarding post-exercise autophagy patterns. The autophagy response appears to depend on exercise intensity, duration, and nutritional status. Higher intensity exercise and fasting conditions typically trigger stronger autophagy responses. While resistance exercise’s effects on autophagy are less studied, research indicates it may actually decrease autophagy temporarily in both young and older adults (He et al., 2012a; Mejías-Peña et al., 2017; Wang et al., 2022). Long-term exercise training’s impact on autophagy shows varied results across studies. Some research found no significant changes in autophagy-related proteins after training periods ranging from 5 days to 3 months, while others observed increased expression of key autophagy regulators after 4–8 weeks. The response appears to vary by muscle fiber type and age, with older mice showing different adaptations compared to younger ones. Exercise-induced autophagy has been documented in various organs including muscle, liver, heart, pancreas, adipose tissue, and even the brain, potentially contributing to exercise’s neuroprotective benefits. While current evidence suggests autophagy plays a crucial role in both immediate exercise response and long-term adaptations, more research is needed to fully understand how different exercise variables and individual factors influence this process (Wang et al., 2022; Wu et al., 2019; Zeng et al., 2020).

### 4.1 Molecular mechanisms of exercise-induced activation of autophagy

When muscles contract during exercise, they experience energy stress similar to nutrient deprivation, triggering changes in cellular messengers like calcium, AMP, NAD+, and reactive oxygen species (ROS). These changes initiate signaling pathways that create a two-phase autophagy response to restore balance. In normal conditions, mTOR and protein kinase A suppress autophagy by inhibiting the induction complex. However, during exercise, when energy demands exceed supply, rising AMP levels activate AMPK while suppressing mTOR. Exercise-induced increases in ROS and NAD + also stimulate AMPK and other stress response proteins like SIRT1 and p38 MAPK, which then trigger autophagy mechanisms. The initial phase of autophagy involves the ULK1 complex, which includes several key proteins working together to form phagophores (He et al., 2012b; Tam and Siu, 2014). During exercise, AMPK activates ULK1 through direct phosphorylation while reduced mTOR activity removes inhibitory signals. Studies with genetically modified mice lacking AMPK-α2 have demonstrated AMPK’s crucial role in exercise-induced autophagy. The next phase involves forming and expanding the autophagosome membrane. This complex process draws membrane components from various cellular sources, including mitochondria, endoplasmic reticulum, plasma membrane, and nuclear envelope. The specific source may vary depending on the trigger and the material targeted for degradation. The beclin-1 complex plays a vital role in autophagosome formation, with Bcl-2 acting as a regulator. Research has shown that exercise activates autophagy by affecting the Bcl-2-beclin-1 interaction (Feng et al., 2023; Park et al., 2023). Studies using various autophagy-deficient mouse models have demonstrated that proper autophagy function is essential for exercise performance and metabolic adaptations. The expansion of autophagic vesicles involves Atg9 and two protein conjugation systems that help form the phagophore. This process culminates in the attachment of LC3 and related proteins to the membrane structure. The importance of these mechanisms is evidenced by studies showing that the loss of key genes in this process can be lethal or severely impact muscle function. Exercise-induced autophagy occurs in multiple tissues, including liver, heart, pancreas, adipose tissue, and brain. However, more research is needed to fully understand how this multi-organ response contributes to exercise benefits. The process concludes with the fusion of autophagosomes with lysosomes, resulting in the breakdown and recycling of cellular components, which can then be used to restore energy balance or build new cellular structures (He et al., 2012b; Sanchez et al., 2014; Zhang and Chen, 2018).

## 5 Exercise-induced autophagy in aging and aged-brain

### 5.1 Physical activity and aged brains

Physical exercise encompasses planned, structured, and repeated activities aimed at enhancing physical fitness, as described by Caspersen. Throughout human evolution, exercise played a crucial role in survival activities like hunting, environmental adaptation, and brain development, showing concurrent evolution of neuroplasticity pathways (Raichlen and Polk, 2013; Wallace et al., 2018). Exercise benefits manifest across different life phases. During pregnancy, controlled moderate exercise helps reduce prenatal depression and can lead to shorter labor duration. Babies born to exercising mothers show enhanced auditory discrimination abilities. These findings support the need for public health initiatives promoting active lifestyles during pregnancy. For teenagers and young adults, exercise enhances attention, cognitive abilities, and helps alleviate depressive symptoms. Older adults benefit from various exercise forms, including Pilates, Tai Chi, resistance training, combined exercise programs, and aerobic activities. While these methods show similar cognitive benefits, improvements may require extended practice periods (Bailey et al., 2018; de Greeff et al., 2018; Jonasson et al., 2017; Labonte-Lemoyne et al., 2017; Perales et al., 2015; Perales et al., 2016). Health organizations advocate for 150 min of moderate weekly exercise. However, many older adults fall short of these guidelines, with some harboring concerns about exercise safety or necessity. Research indicates that even lower activity levels can reduce mortality risk by 22%, suggesting the potential value of light exercise in improving health outcomes and encouraging progression to more vigorous activities (Arem et al., 2015; Franco et al., 2015; Sparling et al., 2015).

Exercise stands out as a primary defense against age-related conditions. Research demonstrates its positive impact on various disorders including dementia, late-life depression, frailty syndrome, and neurodegenerative diseases like Parkinson’s and Alzheimer’s. While studies examining exercise’s epigenetic effects in older adults continue to emerge, some research suggests that exercise alone may not suffice for certain protective mechanisms. Animal studies reveal multiple neuroprotective pathways activated by exercise, including enhanced neurogenesis, preserved dopaminergic neurons, improved antioxidant capacity, and better autophagy (Cancela et al., 2016; Fernandes et al., 2017; Mañas et al., 2018; Quach et al., 2017; Zanetidou et al., 2017). Exercise also helps maintain synaptic connections, reduces inflammation, and supports mitochondrial function. These benefits vary based on exercise type and intensity. Exercise influences broader physiological aspects including brain blood flow, gut microbiome, hormonal balance, and sleep patterns. Improved cerebral blood flow may help prevent cognitive decline and dementia. While exercise’s impact on gut bacteria shows mixed results, it generally supports digestive health. Growth hormone responses to exercise vary among studies, but exercise generally improves sleep quality in older adults (Andreotti et al., 2020). Exercise stimulates BDNF production, supporting neuronal development, synaptic function, and plasticity. This protein also regulates cellular protein maintenance systems, contributing to synaptic remodeling. Exercise offers comprehensive benefits throughout life, particularly in aging. Its neuroprotective effects depend on exercise type and intensity, improving various aspects of health including brain circulation, gut health, sleep quality, and cognitive function. However, some effects, particularly regarding gut microbiota, growth hormone, and cerebral blood flow, show varying results based on exercise parameters (Jia et al., 2008; Neeper et al., 1995; Park and Poo, 2013; Table 2).

**TABLE 2:** Neuroprotective effects of physical exercise in aging brains.

Study/aspect	Exercise type	Effects/benefits	Mechanisms/findings	References
Neuroprotection in Alzheimer’s models	- Treadmill exercise - Voluntary running	- Decreased glia activation - Lowered amyloid-beta (Aβ) peptide levels	Reduced neuroinflammation and Aβ accumulation, contributing to neuroprotection	(Quach et al., 2017)
Resistance exercise in mice	Resistance physical exercise	- Increased neurogenesis - Decreased loss of dopaminergic neurons - Enhanced antioxidant capacity - Improved autophagy	Activation of multiple synergistic neuroprotective pathways	(Jang et al., 2018b)
Aerobic exercise in rats	Aerobic physical exercise	- Reverted synaptic loss in cortex and hippocampus	Up-regulation of Rho-GTPases facilitating synaptic morpho-functional changes	(Jang et al., 2018b)
Endurance exercise in parkinson’s model rats	Endurance physical exercise	- Promoted neuroprotection - Improved mitochondrial biogenesis - Reduced apoptosis - Decreased pro-inflammatory cytokines and α-synuclein protein	Enhanced mitochondrial function and reduced inflammatory responses, leading to decreased neuronal death	(Tarumi and Zhang, 2018)
Aerobic exercise in ischemic brain injury rats	Aerobic physical exercise	- Blocked glia activation - Prevented neuronal death	Neuroprotective by reducing neuroinflammation and maintaining neuronal integrity	(De La Torre, 2004)
General neuroprotective effects	Various (Treadmill, Running)	- Promotes neuroprotective effects depending on exercise type	Improves cerebrovascular health, enhances antioxidant defenses, and modulates neurotrophic factors	(De La Torre, 2004; Jang et al., 2018b; Leeuwis et al., 2018; Li et al., 2017; Quach et al., 2017; Tarumi and Zhang, 2018)

### 5.2 Autophagy and aged brains

As cells age, their proteins and organelles become increasingly damaged and lose proper function. This deterioration leads to a buildup of defective cellular components, accelerating cell death. Research demonstrates that declining autophagy during cellular aging leads to diminished cell performance and potential death, as cells struggle to maintain healthy proteins and organelles (Cuervo and Macian, 2014; Rubinsztein et al., 2011). Impaired autophagy can contribute to brain cell damage associated with aging and neurodegeneration. Age-related decline in autophagy disrupts nerve cell health, potentially accelerating neurodegenerative conditions. However, physical activity has been shown to stimulate autophagy, potentially helping prevent age-related diseases and slow neurodegeneration. Research involving Atg protein deletion has revealed aging-related issues, including excessive accumulation of dysfunctional organelles, endoplasmic reticulum stress, and mitochondrial problems. However, researchers are still investigating whether reduced Atg levels directly cause age-related autophagy dysfunction. Evidence suggests that overactive TORC1, which inhibits autophagy, may contribute to decreased baseline autophagy. Studies indicate that blocking TORC1 activity may extend lifespan (Batatinha et al., 2019; Levine et al., 2011; Metaxakis et al., 2018; Wu et al., 2015).

### 5.3 Exercise as a regulator of autophagy in the aging brains

Research suggests that engaging in consistent physical activity offers numerous health advantages and effectively triggers autophagy. Studies with mice demonstrated that 8 weeks of treadmill exercise enhanced autophagy-related protein levels, particularly Beclin1. The mechanism behind exercise-induced autophagy appears to involve several steps: exercise reduces cellular ATP/AMP ratios, activating AMPK; this activation suppresses mTOR, which releases ULK1 from inhibition; AMPK further enhances ULK1 through phosphorylation; the activated ULK1 complex then stimulates the PI3K complex, leading to increased production of autophagy-related proteins. Additionally, AMPK activates FOXO transcription factors, boosting LC3-II and Atg protein expression (He et al., 2012b; Jang et al., 2018a; Moreira et al., 2017; Schwalm et al., 2015). Evidence indicates that autophagy activation through exercise varies depending on the specific exercise characteristics, including type, length, and intensity level. Research has shown that swimming activities can combat aging effects by restoring proper autophagy function and normalizing mitochondrial dynamics. Studies in rats demonstrated that 10 weeks of swimming enhanced lysosomal breakdown, autophagy activation, and mitochondrial maintenance in the hippocampus, helping prevent age-related cognitive deterioration. These results suggest that exercise-mediated cognitive preservation in older rats relates to improved hippocampal mitochondrial function, with lysosomal degradation playing a crucial role (Luo et al., 2017; Mooren and Krüger, 2015).

Additional research revealed that mice subjected to 8 weeks of running showed enhanced autophagy pathways and increased lysosome production, suggesting improved brain function. This extended physical activity promoted TFEB movement to the nucleus in the cortex, enhancing the expression of genes linked to autophagy and lysosomal processes. Furthermore, moderate exercise has been shown to help prevent early-stage neurodegeneration in aged rats’ substantia nigra by enhancing autophagy and mitochondrial recycling. Based on current evidence, aging disrupts normal autophagy processes, leading to central nervous system damage. While physical exercise shows promise in preventing or reducing these autophagic disruptions, more research is needed to determine the optimal exercise parameters (type, duration, and intensity) for maximizing beneficial effects on central nervous system autophagy (Almeida et al., 2018; Huang J. et al., 2019).

Studies indicate that regular exercise can reduce the risk of cognitive decline in individuals carrying the APOE-ε4 allele, a major genetic risk factor for AD. Exercise promotes brain-derived neurotrophic factor (BDNF) expression, which supports neuronal survival and synaptic plasticity. Additionally, physical activity has been linked to improved glucose metabolism and reduced amyloid-beta accumulation, both of which are critical in AD pathology (Romero Garavito et al., 2025). Exercise has been shown to enhance motor function and delay disease progression in individuals with PD, including those with genetic mutations such as LRRK2 and PARKIN. Physical activity stimulates dopamine release and neuroplasticity, which can help compensate for neuronal loss. Furthermore, exercise may reduce neuroinflammation and oxidative stress, both of which contribute to PD pathology (Ruiz-González et al., 2021). Research suggests that exercise may have neuroprotective effects in ALS by modulating mitochondrial function and reducing oxidative stress. While excessive physical exertion might accelerate disease progression in some cases, moderate exercise has been associated with improved muscle function and delayed symptom onset (Li et al., 2025). Although ASD is primarily a neurodevelopmental disorder, studies indicate that exercise can improve behavioral outcomes, social interaction, and cognitive function in individuals with ASD. Physical activity has been linked to increased BDNF levels, which may enhance synaptic connectivity and neuroplasticity. Additionally, exercise can help regulate stress responses and improve mood stability (Romero Garavito et al., 2025). Overall, while genetic predispositions play a significant role in these disorders, exercise appears to be a promising non-pharmacological intervention that can support brain health and mitigate disease impact.

## 6 Physical exercise, autophagy, and neurodegenerative diseases

### 6.1 Role of autophagy in neurodegenerative conditions

Neurodegenerative disorders are characterized by the formation of abnormal protein clusters. These include Aβ and APP C-terminal fragments in Alzheimer’s disease, altered α-synuclein in Parkinson’s disease, abnormal huntingtin protein in Huntington’s disease, and defective SOD1 and TDP-43 in ALS. The autophagy-lysosome system typically processes these protein accumulations. Notably, mutations in autophagy receptors (including p62, OPTN, NBR1, and ALFY/WDFY3) are linked to these conditions. The natural aging process, a key risk factor in neurodegeneration, reduces autophagy efficiency (Park et al., 2020). Impaired autophagy is considered a contributing factor in neurodegenerative diseases. Research indicates that enhancing autophagy could be an effective treatment approach. Studies show that activating autophagy, particularly through p62, helps eliminate problematic proteins like mHtt, insoluble tau, and Aβ42. Conversely, blocking autophagy with compounds like 3-MA or bafilomycin A1 leads to increased mHtt aggregates in cellular and animal models (Deng et al., 2017; Menzies et al., 2017; Scrivo et al., 2018; Stavoe et al., 2019; Tsvetkov et al., 2013). AD, the most prevalent neurodegenerative condition, is marked by Aβ deposits and tau tangles in brain tissue. Aβ peptides result from APP processing by various secretases in cellular compartments. Autophagy plays a crucial role in removing Aβ and APP-CTF. Enhanced p62 or TFEB activity reduces Aβ plaque formation and improves disease outcomes in mice. However, increased Aβ oligomers can impair autophagy by disrupting transport and lysosome formation. Studies of AD patients’ brain tissue reveal accumulated autolysosomes containing cathepsin, indicating defective lysosomal protein breakdown. Autophagy-related protein levels often show abnormalities in AD patients (Castellazzi et al., 2019; Cho et al., 2019; Lee et al., 2019; Song et al., 2020; Zhang et al., 2019).

Genetic mutations causing familial AD impact autophagy function. PSEN1, part of the γ-secretase complex, is essential for lysosome maintenance and autophagy gene expression. PSEN1 mutations affect lysosome acidification and autolysosome function. Additionally, PSEN1-deficient neural stem cells show reduced TFEB expression, leading to decreased autophagy-related gene activity (Chong et al., 2018; De Strooper et al., 1998; Lee et al., 2010). PICALM variations have been identified in AD cases. This protein facilitates clathrin-mediated endocytosis of SNAREs and APP. Beyond endocytosis, PICALM influences autophagosome development and maturation through SNARE regulation. It also acts as an autophagy receptor, forming complexes with AP-2 to facilitate APP-CTF degradation. Reduced autophagy in AD patients may also stem from decreased Beclin-1 and VPS35 levels. These proteins regulate APP processing and autophagy function. Studies show that reducing Beclin-1 leads to decreased neuronal autophagy and increased Aβ accumulation. Beclin-1 helps move APP to autophagosomes and influences phagocytosis through VPS35 regulation (Park et al., 2020). Abnormal tau protein degradation is also autophagy-dependent. AD patients’ brain tissue shows accumulation of autophagy-related proteins and lysosomal defects, often coinciding with hyperphosphorylated tau. This modified tau interacts with autophagy receptors like p62, NDP52, and OPTN. PICALM assists in tau breakdown through autophagy. Problems with dynein-dynactin transport can increase tau accumulation, while enhanced autophagy helps reduce tau aggregation (Dolan and Johnson, 2010; Jo et al., 2014; Lee et al., 2019; Piras et al., 2016; Xu et al., 2019).

PD involves progressive movement problems, characterized by α-synuclein-containing Lewy bodies in dopamine neurons. Studies show that removing ATG7 leads to age-related increases in α-synuclein inclusions. While mutated α-synuclein can be cleared by autophagy, its accumulation disrupts various autophagy steps, including omegasome formation, autophagosome transport, and lysosomal enzyme function. PD shows altered expression of autophagy genes, with decreased TFEB-mediated transcription during advanced stages. The A30P α-synuclein variant increases ZKSCAN3 activity, affecting LC3 and p62 expression. Increasing TFEB levels improves PD symptoms and reduces α-synuclein buildup (Arotcarena et al., 2019; Hoffmann et al., 2019; Lei et al., 2019; Moors et al., 2019; Singleton et al., 2003; Yan et al., 2018). GBA mutations represent a major genetic risk for PD. These mutations affect protein levels and enzyme activity, disrupting lysosomal function. Early-stage PD shows decreased GBA activity alongside increased α-synuclein accumulation. A feedback loop exists where accumulated α-synuclein further impairs GBA function. LRRK2 mutations also significantly contribute to PD risk. Their role in autophagy remains debated, as both loss and gain of function mutations impair autophagy-lysosome pathways. Some LRRK2 mutations affect vesicle trafficking and lysosomal function. ATP13A2 mutations, linked to early-onset PD, affect lysosomal pH regulation. These mutations impact TFEB activity and autophagosome-lysosome fusion. ATP13A2 dysfunction leads to α-synuclein accumulation, contributing to PD pathology. VPS35 mutations may also contribute to PD-related autophagy defects. Decreased VPS35 expression appears in PD patients’ substantia nigra. The D620N mutation affects ATG9A distribution and impairs autophagy function (Esteves and Cardoso, 2017; García-Sanz et al., 2017; Wallings et al., 2019; Wang R. et al., 2019; Zavodszky et al., 2014).

### 6.2 Exercise and autophagy in neurological diseases

Research by Kim and colleagues revealed that exercise can enhance autophagy responses. Studies found that running on treadmills triggers autophagy in the brain’s cortex, which plays a key role in exercise-related metabolic advantages in mature mice. Evidence suggests that pre-exercise conditioning combined with p38 inhibition can shield neurons during stroke by controlling autophagy levels. Physical activity has been shown to enhance neurological outcomes by reducing cell death, stimulating new neuron growth, and lessening autophagosome buildup near stroke-affected areas in rat studies. Research demonstrates that treadmill exercise beforehand protects brain tissue by limiting stroke damage through autophagy regulation, specifically by increasing phosphorylated ERK1/2 and restoring p62 levels (Betin and Lane, 2009; Kim et al., 2013; Zhang et al., 2013; Zhang et al., 2014).

Pre-exercise conditioning may prevent excessive exercise damage by triggering mitophagy—recruiting LC3 to move Bnip3 to mitochondria, a process activated by H_2_O_2_ and influenced by Beclin1-dependent autophagy. Regular moderate exercise appears to enhance both mitophagy and autophagy, potentially helping prevent early substantia nigra deterioration. Exercise training has been found to counteract reduced autophagic activity in elderly subjects’ blood cells by adjusting p62 protein levels, LC3II/I ratios, and various autophagy regulators including Atg12, Atg16, beclin-1, and phosphorylated ULK-1. Dagon’s research indicates exercise induces autophagy through AMPK-ULK1/Atg1 activation. An 8-week resistance training program was found to stimulate autophagy while reducing inflammation and cell death in elderly subjects’ blood cells. Swimming exercise appears to improve mitochondrial dynamics and regulate autophagy by reducing miR-34a, potentially slowing aging processes. Novel findings by Ogura revealed exercise causes two-phase autophagy changes—an initial LC3II decrease followed by an increase after 1 h, possibly connected to mTOR regulation. In Alzheimer’s disease models, aerobic exercise improved irregular autophagy by reducing mTOR expression (Almeida et al., 2018; Fucà et al., 2017; Jang et al., 2018a; Mejías-Peña et al., 2016; Mejías-Peña et al., 2017; Yuan et al., 2018; Zhao et al., 2018; Table 3).

**TABLE 3:** Influence of physical exercise on autophagy in neurodegenerative diseases.

Model	Intervention	Outcomes/results	Autophagy mechanisms/proteins involved	Neurodegenerative disease	References
Adult mice	Exercise training	- Promoted autophagy response - Regulated metabolic benefits	Activation of autophagy pathways	Various	(Kim et al., 2013)
Mice, rats	Moderate-to-high intensity treadmill training	- Induced autophagy in cerebral cortex - Enhanced metabolic efficiency - Increased Aβ clearance and reduced plaque burden - Improved cognitive and motor functions	Upregulation of LC3-II, Beclin-1, PGC-1, SIRT1 Downregulation of mTOR	Alzheimer’s disease, PD	(Almeida et al., 2018; Fucà et al., 2017; He et al., 2012b; Herring et al., 2016; Koo and Cho, 2017; Kou et al., 2017; Mejías-Peña et al., 2016; Ogura et al., 2011; Yuan et al., 2018; Zhang et al., 2013; Zhang et al., 2014; Zhao et al., 2018)
Rats	Treadmill training preconditioning	- Provided neuroprotective effects - Inhibited autophagosome accumulation - Alleviated neuronal injuries following ischemic stroke	Suppression of p38 MAPK, reduced autophagosome accumulation	Cerebral ischemic stroke	(Zhang et al., 2014; Zhang et al., 2013)
Rats	-	- Inhibited exhaustive exercise injuries - Promoted mitophagy - Increased LC3 and Bnip3 translocation to mitochondria	Induction of mitophagy via LC3 and Bnip3	Stroke-like Injury	(Yuan et al., 2018)
Mice	Moderate aerobic exercise	- Improved mitochondrial biogenesis - Enhanced autophagic flux - Increased Aβ clearance - Reduced plaque burden	Increased expression of PGC-1, SIRT1 Enhanced LC3-II/I ratio Upregulated Beclin-1	Alzheimer’s disease	(Almeida et al., 2018)
Elderly humans	Regular exercise training	- Downregulated p62 expression- Upregulated LC3-II/I ratio - Altered levels of Atg12, Atg16, Beclin-1, p-ULK1	Enhanced autophagic flux, regulation of autophagy-related proteins	Various	(Mejías-Peña et al., 2016)
Mice	Treadmill training	- Decreased Aβ and tau aggregates - Improved memory performance - Mitigated autophagy dysfunction - Reduced oxidative injury and inflammation	Upregulation of autophagy markers, improved lysosomal function	Alzheimer’s disease	(Zhang et al., 2014; Zhao et al., 2018; Herring et al., 2016)
PD mouse models	Treadmill exercise	- Improved motor function - Alleviated dopaminergic neuronal cell death - Enhanced autophagic flux - Promoted α-synuclein clearance	Enhanced LC3-II levels, upregulated autophagic flux	Parkinson’s disease	(Fucà et al., 2017; Jang et al., 2018a; Koo and Cho, 2017)
Tambaleante mice	Motor training	- Increased BDNF expression - Alleviated autophagic flux - Slowed neurodegeneration	Upregulation of BDNF, enhanced autophagy	Various neurodegenerative diseases	(Fucà et al., 2017)

Exercise stimulates AMP-activated protein kinase (AMPK), which promotes autophagy via ULK1 phosphorylation. Physical activity reduces mTOR signaling, a known suppressor of autophagy. Exercise may enhance transcription factor EB (TFEB) activity, increasing lysosomal biogenesis and autophagy flux. Exercise may modulate forkhead box O3 (FoxO3), which influences autophagy-related gene expression. Several studies have investigated the expression of autophagy-related markers in human tissues following exercise interventions. Beclin-1 and LC3-II levels have been shown to increase in response to exercise preconditioning, suggesting enhanced autophagic flux. p62 expression varies depending on exercise intensity, with exhaustive exercise leading to transient increases followed by clearance (Huang et al., 2021). Ischemic post-conditioning studies have demonstrated upregulation of LC3-II and Beclin-1 in the hippocampus, indicating neuroprotective effects (Huang L. et al., 2019). These findings highlight the potential of exercise to modulate autophagy in humans, but more tandardized protocols are needed to establish consistent biomarker responses.

The type, intensity, and duration of exercise significantly influence autophagic responses. Endurance exercise has been linked to increased SQSTM1/p62 expression, suggesting enhanced autophagic clearance. Resistance training appears to regulate LC3-II levels, with long-term resistance exercise promoting sustained autophagy activation. Concurrent training (combining endurance and resistance) may optimize autophagic flux by balancing mitochondrial turnover and protein degradation. Understanding these modality-specific effects will help tailor exercise interventions for neurodegenerative disease prevention (Kwon et al., 2018; Pinto et al., 2021). Long-term studies are crucial to establish whether exercise-induced autophagy translates into cognitive improvements and neuroprotection. PACAP–Sirtuin3 activation has been shown to alleviate cognitive impairment through autophagy modulation in Alzheimer’s disease models. DHA supplementation trials have demonstrated enhanced autophagic clearance of amyloid-beta, leading to improved cognitive function in elderly individuals. Multi-targeted autophagy strategies combining exercise with pharmacological interventions have been proposed to mitigate brain aging and neurodegeneration. These findings suggest that autophagy activation via exercise could be a viable strategy for aging-related cognitive decline, but further longitudinal human trials are needed to confirm these effects (Wang et al., 2023; Zhang Y. et al., 2018). To enhance the translational impact of the review, the authors should incorporate human studies that measure autophagy biomarkers post-exercise, explore modality-specific effects, and highlight longitudinal trials linking autophagic flux to cognitive benefits. This approach will provide a comprehensive framework for understanding exercise-induced autophagy in aging and neurodegenerative diseases.

### 6.3 Exercise-mediated autophagy in neurodegenerative diseases

Research has demonstrated that regular exercise enhances mitochondrial performance, leading to better oxidative metabolism and reduced oxidative stress. Recent studies highlight exercise’s crucial role in stimulating autophagy, producing beneficial effects across various tissues. Studies in mice have revealed that exercise training enhances the expression of several genes, including PGC-1, SIRT1, and citrate synthase, while increasing mitochondrial DNA across brain regions, suggesting enhanced mitochondrial formation. Studies comparing different exercise types found that endurance training, rather than casual physical activity, enhanced autophagy, mitophagy, and mitochondrial function in rats’ brain regions, correlating with improved movement and exploratory behavior. Regular physical activity has been linked to decreased risk of neurological disorders, with aerobic exercise promoting new neuron formation in both young and aged rodents. While the exact mechanisms remain under investigation, research indicates that elevated reactive oxygen species (ROS) in neural stem cells promote self-renewal and neurogenesis, with acute exercise shown to boost ROS production in specific brain regions (Ahlskog et al., 2011; Marques-Aleixo et al., 2015; Radák et al., 2013; Steiner et al., 2011).

Exercise shows promising effects on Alzheimer’s and Parkinson’s diseases by increasing brain plasticity, improving amyloid-beta (Aβ) removal, and reducing inflammation and oxidative damage, resulting in enhanced cognitive function. Exercise training also improves systemic markers of neurodegeneration by reducing cellular aging and improving muscle function. Autophagy plays a vital role in glial cell function, particularly in microglia—the brain’s immune cells that eliminate pathogens, dead cells, and proteins like Aβ. Research has shown that microglia lacking autophagy-related proteins struggle to clear Aβ aggregates, and Alzheimer’s patients’ microglia show reduced levels of these proteins. Elevated Aβ levels trigger inflammatory responses in microglia, while autophagy helps regulate these inflammatory pathways. Studies demonstrate that both voluntary running and structured treadmill training reduce Aβ accumulation and excessive microglial activation in aged mice and Alzheimer’s models. Exercise may enhance microglial autophagy, potentially improving Aβ clearance and reducing inflammation, though direct evidence is still needed. Recent research shows that moderate treadmill exercise can activate autophagy in specific brain regions during early neurodegeneration (Batatinha et al., 2019; He et al., 2017; Herring et al., 2016; Lauzé et al., 2016; Ye et al., 2017; Zhang Y. et al., 2018). The regulation of autophagy and mitophagy shows promise as an anti-aging strategy. Exercise effectively activates these processes, helping prevent age-related immune decline and associated conditions. However, more research is needed to fully understand how exercise modulates autophagy across different cell types, which could lead to new therapeutic approaches for age-related diseases.

Autophagy, the cellular process responsible for degrading misfolded proteins and damaged organelles, is crucial in maintaining neuronal health. Dysregulated autophagy contributes to the pathogenesis of AD, PD, HD, and ALS by impairing the clearance of toxic protein aggregates characteristic of each disease. In AD, impaired clearance of autophagic vacuoles (AVs), rather than a failure to induce autophagy, leads to their accumulation, which is linked to mutations in presenilin 1 (PS1) affecting lysosomal function. In ALS, specific gene expression changes related to autophagy occur following exercise, and aerobic exercise has been shown to modulate autophagy-related proteins (LC3B-I and LC3B-II), glucose metabolism, and disease progression in ALS mouse models. Although autophagy dysfunction is implicated across these diseases, the precise molecular pathways and their impact on disease progression differ, reflecting disease-specific pathologies and genetic factors. Exercise generally shows neuroprotective effects across these diseases but with notable differences. In AD, exercise reduces amyloid-beta accumulation, improves neurotrophin levels (e.g., BDNF), and delays hippocampal aging-related gene expression changes. In PD, exercise improves motor function and dopaminergic neuron survival, with combined aerobic and strength training showing benefits in insulin sensitivity and cognitive function. In HD, exercise delays motor deficits and increases BDNF expression, improving striatal function. In ALS, moderate-intensity exercise (e.g., swimming) delays motor deficits and disease progression, whereas high-intensity exercise may accelerate it, indicating a need for tailored exercise prescriptions. Exercise also influences insulin resistance differently among these diseases, with mixed exercise models more effective in PD and swimming/resistance exercise improving insulin signaling in AD models (Guo et al., 2018; Guo et al., 2022; Memon et al., 2020; Meng et al., 2019; Shen et al., 2024).

## 7 Potential for integrating exercise into therapeutic strategies for neurodegenerative diseases

Exercise stands out as a primary non-pharmacological approach for treating various health conditions. Regular physical activity helps prevent or slow the progression of age-related conditions, including bone loss, muscle deterioration, and metabolic and cardiovascular issues. Research indicates that exercise can also combat neurodegenerative disorders characterized by amyloid accumulation. While these conditions differ in their origins and symptoms, they share common features like neurotoxicity and protein aggregate formation. The underlying mechanism typically involves protein misfolding, leading to toxic accumulations that cause progressive neuron loss in specific brain regions (Cariati et al., 2021a; Cariati et al., 2021b; Diociaiuti et al., 2021; Myers et al., 2019). AD, marked by memory decline and cognitive deterioration, results from toxic accumulations of Aβ protein and phosphorylated Tau. As the leading cause of dementia affecting approximately 30 million people globally, AD’s prevalence is projected to double in Europe and triple worldwide by 2050, emphasizing the urgent need for effective interventions. Exercise shows promise as both a preventive measure and treatment for AD, likely due to its ability to enhance brain blood flow, expand hippocampal volume, and promote new neuron formation (Cass, 2017; Scheltens et al., 2021; Villain and Dubois, 2019). Recent research demonstrates that moderate exercise improves cognitive function in AD mouse models, with exercising mice showing better exploratory behavior and reduced cellular abnormalities compared to sedentary controls, studies support these findings. For instance, research involving AD patients showed that regular aerobic exercise improved functional capacity, memory performance, and hippocampal volume, suggesting exercise’s protective effects against early-stage AD damage (Hwang et al., 2022).

While the exact mechanisms remain under investigation, exercise-induced neurotrophins appear crucial. Brain-derived neurotrophic factor (BDNF) has been shown to counter memory loss in animal models. AD-related Aβ aggregates reduce BDNF expression through complex cellular pathways, affecting memory and learning processes. In Parkinson’s disease, characterized by α-synuclein accumulation and dopamine neuron loss, exercise remains the most effective primary prevention strategy. Studies show that resistance exercise in PD models reduces toxic protein accumulation and inflammation markers. Human trials demonstrate that high-intensity exercise particularly benefits PD patients, resulting in fewer motor function changes compared to standard care. The relationship between exercise, neurotrophins, and cellular cleaning mechanisms (autophagy) offers promising therapeutic potential for neurodegenerative conditions. Exercise stimulates the production of various neurotrophic factors that promote neuron survival and brain health while enhancing cellular cleanup processes (Bonanni et al., 2022). However, additional research is needed to fully understand these mechanisms and develop targeted interventions for individuals affected by or at risk for these disorders (Table 4).

**TABLE 4:** Therapeutic integration of exercise in neurodegenerative diseases.

Disease	Model	Intervention	Outcomes/results	Mechanisms/neurotrophins involved	References
Alzheimer’s disease (AD)	Mouse (transgenic AD model)	Moderate-intensity treadmill exercise 50 min/day, 5 days/week	- Increased exploration of new objects in exercised mice - Reduced expression of apoptotic death factors in trained mice	- Reversal of cellular abnormalities caused by Aβ deposition - Potential increase in BDNF and other neurotrophins	(Hwang et al., 2022)
	Human (76 individuals with early AD)	Aerobic exercise 150 min/week vs. stretching controls	- Improved functional capacity - Enhanced cardiorespiratory fitness - Improved memory performance - Increased bilateral hippocampal volume	- Enhanced cerebral blood flow - Increased hippocampal volume and neurogenesis	(Morris et al., 2017)
	AD Rats	Transplantation of BMSCs overexpressing NT-3	- Improved cognitive function - Promoted neurorigeneration	- Activation of β-catenin pathway - Enhanced NT-3 expression	(Yan et al., 2021)
	AD Rats	Grafting fibroblasts modified with NT-4 gene into hippocampus	- Increased survival of cholinergic neurons - Preservation of learning and memory functions	- Overexpression of NT-4 - Neuroprotection of hippocampal neurons	(Liu et al., 2009)
Parkinson’s disease (Kleinloog et al., 2022)	Mouse (MPTP-induced PD model)	Resistance exercise	- Preserved motor function similar to control - Lower levels of α-synuclein - Reduced expression of TLR2 and NF-κB	- Reduction of neurotoxic α-synuclein aggregates - Decreased neuroinflammation	(Jang et al., 2017)
	Human (128 PD subjects)	High-intensity treadmill vs. moderate-intensity vs. control	- High-intensity exercise group showed fewer motor changes compared to control - Met non-futility threshold	- Increased expression of BDNF and GDNF - Enhanced neurotrophic support	(Schenkman et al., 2018)
	Animal (PD model)	Oral administration of gemfibrozil	- Improved motor activities - Increased transcriptional activity of GDNF gene in astrocytes	- Upregulation of GDNF expression - Neuroprotection of dopaminergic neurons	(Gottschalk et al., 2021)
	Rat (6-OHDA-treated PD model)	Transplantation of rNSC-NT3	- Improved spatial learning ability - Protection of dopamine neurons in substantia nigra - Reversal of main PD symptoms	- Enhanced NT-3 expression - Activation of Wnt/β-catenin signaling pathway	(Gu et al., 2009)
	Embryonic rat midbrain floor	Synergistic administration of NT-4 and GDNF	- Protection of dopaminergic neurons from oxidative stress-induced damage	- Synergistic action of NT-4 and GDNF - Reduction of oxidative stress-related neurodegeneration	(Lingor et al., 2000)

Exercise induces autophagy primarily through cellular energy-sensing pathways, notably the AMPK-ULK1-mTOR axis, which promotes autophagy-related protein expression and lysosomal biogenesis. This process appears to be modulated in an exercise type-, intensity-, and duration-dependent manner. Animal studies show that moderate-intensity treadmill running (e.g., 8 weeks, 5 days/week) or swimming can activate autophagy markers (e.g., LC3-II, Beclin1), improve mitochondrial quality control, and enhance lysosomal degradation in the brain, thereby preventing age-related cognitive decline and neurodegeneration. In Parkinson’s disease models, mild-to-moderate treadmill exercise for 8 weeks improved motor function and reduced α-synuclein accumulation via autophagy activation. Human exercise guidelines for PD recommend moderate-intensity exercise 3–5 days per week, starting with 20 min per session and progressing to 60 min, with activities tailored to patient preference (e.g., walking, ergometry, aquatic exercise). Other forms of exercise such as swimming have also been shown to rescue autophagy function and mitochondrial dynamics in aged rodents, suggesting that aerobic and endurance exercises are beneficial for autophagy induction. Despite promising animal data, there is a paucity of human studies directly measuring exercise-induced autophagy changes in neurodegenerative diseases. Consequently, no universally accepted protocols exist for exercise prescription specifically targeting autophagy optimization. Current recommendations emphasize moderate-intensity aerobic exercise with gradual progression in duration and frequency, customized to individual capacity and disease status, but these are based more on clinical symptom improvement than direct autophagy biomarkers. While exercise is advocated to induce autophagy and confer neuroprotection, precise recommendations on exercise type, intensity, and duration remain to be clearly defined. Evidence favors moderate-intensity aerobic exercise (e.g., treadmill running, swimming) performed regularly (3–5 times per week, 20–60 min per session) to activate autophagy pathways. Future research is needed to establish optimized, disease-specific exercise protocols that directly target autophagy regulation for maximal therapeutic benefit (Andreotti et al., 2020; Cart and Pauling, 2025; Goenawan et al., 2023; Li et al., 2023; Zhou et al., 2025).

## 8 Future directions and conclusion

The growing body of evidence underscores the critical role of physical exercise in promoting brain health and mitigating the effects of neurodegenerative diseases such as Alzheimer’s and Parkinson’s. Exercise not only enhances cognitive function and neuroplasticity but also reduces the accumulation of neurotoxic proteins and inflammation, thereby protecting neuronal integrity. The involvement of neurotrophic factors, which are upregulated through physical activity, highlights a promising pathway for therapeutic interventions aimed at preserving cognitive functions and preventing disease progression. Given the increasing prevalence of neurodegenerative disorders, integrating exercise into treatment plans represents a viable strategy for enhancing patient outcomes and improving quality of life. Future dimensions of this field should focus on elucidating the precise molecular mechanisms through which exercise influences autophagy and neurotrophic factor expression. Longitudinal studies examining the long-term effects of different types and intensities of exercise on brain health across diverse populations will be essential. Additionally, exploring the synergistic effects of exercise with pharmacological therapies could lead to more comprehensive treatment approaches. Investigating personalized exercise regimens tailored to individual genetic and phenotypic profiles may further enhance therapeutic efficacy. Finally, public health initiatives aimed at promoting physical activity as a preventative measure against neurodegeneration should be prioritized to address this growing health crisis effectively.
